# Surgery for colorectal liver metastases

**DOI:** 10.1038/sj.bjc.6605659

**Published:** 2010-04-27

**Authors:** J N Primrose

**Affiliations:** 1 University Surgical Unit, University of Southampton, Southampton General Hospital, Mailpoint 816, Tremona Road, Southampton SO16 6YD, UK

**Keywords:** colorectal liver metastases, surgery, chemotherapy, minimally invasive surgery, portal vein embolisation

## Abstract

In this review the surgery of colorectal liver metastases is discussed. It has long been known that liver surgery can cure metastatic colorectal cancer although in only a small proportion of the population with the disease. However with better understanding of the natural history of the condition and advances in technique more patients can have safe, potentially curative surgery. The multidiscipline management of patients with effective chemotherapy has led to more patients benefiting from surgery after reducing the size of the metastases and allowing operation on patients who were previously inoperable. Chemotherapy also improves at least the medium-term outcome in those who are operable at the outset. Minimally invasive techniques have been developed so that major hepatectomy may be accomplished in up to half of such cases with a very short hospital stay and limited interference with quality of life. Lastly, using portal vein embolisation to cause hypertrophy of the future liver remnant and on occasions combining it with staged liver resection allows potentially curative surgery on patients who previously could not have survived resection. These developments have led to more patients being cured of advanced colorectal cancer.

No randomised trial has ever been performed to show that patients with colorectal liver metastasis from cancer can be cured by liver surgery. That being said the abundance of clinical evidence suggests that cure may often follow surgical treatment. Our systematic review (Simmonds *et al*, 2006) shows that around 30% of patients will achieve 5-year survival after liver resection and, although relapse still occurs between 5 and 10 years, 20% of the population will still be alive at that point. Further relapse seems not to occur after 10 years (Tomlinson *et al*, 2007; Rees *et al*, 2008). Accepting the highly selective nature of the population, this impressive cancer control is not observed in patients who do not have resectional surgery, even those with indolent disease (Goslin *et al*, 1982). Indeed a recent epidemiological study in the UK showed that the outlook of patients with colorectal liver metastases who went on to resection was no different than in patients in stage-III (Dukes's C) (Morris *et al*, 2009).

## Prognostic factors

The prognostic factors are largely established; patients with four or more metastases have a worse outlook as do patients with larger metastases and where the primary tumour is poorly differentiated or has lymph node involvement. Extra-hepatic disease and a high CEA are also adverse features and after operation a positive resection margin has prognostic significance. These factors emerge from most of the studies that have been undertaken (Simmonds *et al*, 2006; Rees *et al*, 2008). At present liver resection is the only modality shown to convincingly cure liver metastasis, although ablative treatments are currently showing some promise (Mulier *et al*, 2008).

## Indications and resectability

Central to any discussion of techniques of liver resection are the linked issues of indications and resectability. It is clear from experience supported by the evidence cited above (Simmonds *et al*, 2006; Rees *et al*, 2008) that excellent results can be obtained with liver resection by employing highly selective criteria. Most liver surgery units though will offer surgery on a range of presentations accepting that the outlook in terms of cure will vary. Further, indications have changed greatly in recent years as it has been shown that good results can be achieved in patients previously thought incurable with effective chemotherapy (Adam *et al*, 2009).

The current standard approach is that resection is indicated provided the disease can be resected surgically (including resectable extra hepatic disease), with sufficient residual liver to enable the patient to survive while the liver regenerates (Simmonds *et al*, 2006; Rees *et al*, 2008; Adam *et al*, 2009). Indeed the unique regenerative power of the liver allows major hepatectomies to be performed repeatedly, with the liver regenerating between operations (Wicherts *et al*, 2008).

**Figure 1:**
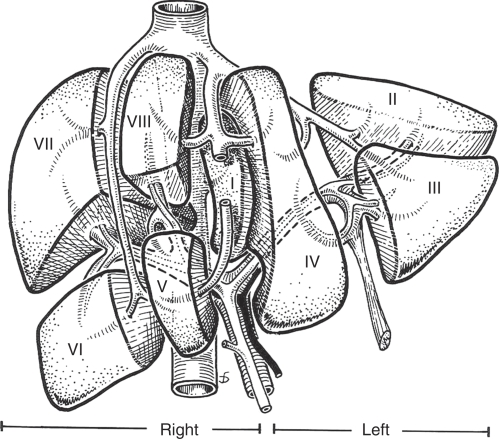
The segments of the liver as first described by Couinaud *et al* (1957).

Determining resectability requires detailed knowledge of liver anatomy. Surprisingly, liver anatomy was not widely understood until the mid twentieth century. Even in the most detailed anatomical text books originating in the English-speaking world the liver appears little more than a box into which vessels enter and leave but without any internal anatomy. Couinaud *et al* (1957) in France first described the segmental anatomy of the liver, which is essential to modern liver surgery. In essence the liver has four sectors and eight segments (Figure 1). Each segment has a portal vein, hepatic artery, and bile duct and hepatic vein branches. This means they can all be resected separately leaving for the most part the other segments of the liver uncompromised.

Resectability is much debated by liver surgeons, but in essence the current consensus would be that after resection the patient should have at least two liver segments remaining and in continuity. The remnant must be supplied by a portal vein and a hepatic artery, and there must be a bile duct, which is or can be made to be in continuity with the gut. In addition one of the three main hepatic veins should remain. Furthermore, the remnant should be around 20–25% of the total functional liver volume (Hemming *et al*, 2003). This remaining liver volume is calculated on the basis of the pre-operative axial imaging and is termed the future liver remnant (FLR). There are some caveats. Survival with an FLR of 25% requires the residual liver to be free from disease such as cirrhosis, steatosis and nowadays, most importantly, chemotherapy-associated steatohepatitis (CASH) (Fong and Bentrem, 2006). Patients with a diseased liver require a larger remnant to survive. However, an inadequate FLR is also not now an absolute contra-indication for surgery. Techniques are available to ‘grow’ liver to facilitate resection and these are considered further below.

## Use of cytotoxic chemotherapy

Surgical treatment of liver metastasis has undoubtedly been transformed by cytotoxic chemotherapy and more recently biological agents. The first indications of the versatility of chemotherapy came with the observation that the combination of fluoropyrimidine and oxaliplatin could convert patients with irresectable disease to resectable with a reasonable degree of frequency (Giacchetti *et al*, 1999). Furthermore, the outcome after resection in such patients was no different from patients who were up-front resectable. This observation has been repeated many times and is now widely accepted. Indeed there is very good correlation between the resectability rate in patients considered inoperable at presentation and the response rate to the therapeutic schedule (Folprecht *et al*, 2005). Indeed, conversion to operability is now being used as a surrogate end point in trials on the treatment of advanced colorectal cancer. This is a sensible approach; resectability and 5-year survival are closely related (Folprecht *et al*, 2005; Simmonds *et al*, 2006). The results of the recently published EPOC trial (Nordlinger *et al*, 2008) adds evidence to support of the routine use of chemotherapy in all patients by showing an 8% improvement in PFS at 3 years who were operable at the outset.

## Liver imaging

Successful metastasis surgery requires accurate assessment of the anatomical localisation of the disease both within and without the liver. Contrast CT of the chest, abdomen and pelvis is commonly used as the initial imaging modality, followed by contrast MRI for those which appear to be resectable. MRI probably has higher levels of sensitivity for small metastases and clearly it is essential that all the disease within the liver is adequately documented (Rappeport and Loft, 2007). Various systems are available to produce three-dimensional reconstructions of the liver as well as volumetry to assess the FLR. For the most part, however, surgeons become used to integrating the 2D images to a three-dimensional surgical plan. What is essential is that the relationship of the metastatic disease to the crucial structures in the liver is appreciated. Surgeons generally prefer good margins around vital structures, preferably 10 mm or more. However, it has been known for a while that any margin at all will actually suffice as most liver metastases are ‘pushing’ rather than infiltrative (de Haas *et al*, 2008).

PET/CT is commonly used for patients with liver metastases being considered for liver resection, but its role is probably to detect extra-hepatic disease (Finkelstein *et al*, 2008). A randomised trial shows that the incidence of a futile laparotomy (incomplete removal of tumour or disease-free survival of less than 6 months) is reduced from 45 to 28% without significantly adversely affecting the disease-free or overall survival (Ruers *et al*, 2009). Laparoscopy with or without intra-operative ultrasound has also been used in preoperative assessment although in recent years has been used selectively (Pilkington *et al*, 2007). This is partly related to the morbidity associated with the procedure (4% major complication rate) and the fact that peritoneal disease, often detected at laparoscopy, may also be detected non-invasively by CT-PET.

## Surgical margins and the ‘disappearing’ metastasis

Effective chemotherapy converts inoperable patients to operable ones, principally because tumour shrinkage produces margins on vital structures. There is much debate about how to manage a patient who has responded well or even completely to chemotherapy. Is the margin that is produced by shrinkage of the metastases really tumour-free? Experience in fact suggests that it is (de Haas *et al*, 2008), and this is different from other malignancies such as breast cancer. The next question is what to do about the disappearing lesion that can no longer be resolved on imaging. Here the situation is more difficult. Evidence suggests that most such lesions are not completely ‘sterilised’ and will grow again if left untreated (Benoist *et al*, 2006). For this reason it is optimal to remove all the liver that has ever contained a tumour. However, sometimes this is undesirable or even impossible. These circumstances increasingly involve a wait-and-see approach being adopted. The ‘missing’ lesions are looked for on serial imaging and if they reappear re-resection or other ablation techniques may be used.

## Operative technique

The operative technique for metastecetomy has become increasingly refined, and with this refinement has come safety. The best evidence to support this has come from multi-centre trials. One such large multi-centre trial with obligate prospective registration showed that in patients with up-front resectable disease and four metastases or less the 30-day mortality from any major liver resection is less than 1% (Nordlinger *et al*, 2008). This is lower than for instance the operative mortality associated with colorectal resection (Guillou *et al*, 2005). The principal feature in reducing complications and mortality is almost certainly lowering blood loss, although most data actually come from studies of hepatocellular cancer (Pamecha *et al*, 2009). It may not be just directly due to the effects of blood loss or lack of its effect on short-term outcome, but possibly because precise careful surgery associated with low blood loss is also good surgery. Using modern techniques most patients having a straightforward liver resection will not require blood transfusion and cross-matching of blood is now no longer routine.

It is important to realise that liver surgery is a team approach and the anaesthetist is a crucial member of the team. Blood flow into the liver can be easily controlled by occluding the inflow in the free edge of the lesser omentum where the portal vein and hepatic arteries run. Therefore bleeding from the liver with a clamp on the inflow can only be from the hepatic veins, which are in continuity with the vena cava without any intervening valves. Thus if the central venous pressure and the pressure in the vena cava is low and the liver is mobilised and brought up into the wound even then an opening in a hepatic vein should not result in bleeding (Wang *et al*, 2006). This can be shown to be so in practice. If a large vein is opened inadvertently, very often the blood simply oscillates in the open vein because the pressure gradient between the vein and the air is essentially zero. This allows, essentially, bloodless surgery. Very often complete in-flow occlusion is not actually needed and certainly not to the FLR. For instance if a right hepatectomy is being performed it is normal to divide the inflow to the right liver either extra-hepatically or intra-hepatically (Cresswell *et al*, 2009). This has the effect of depriving the right liver of most of its blood supply as there is very little crossover. Once the inflow has been dealt with it is usual to divide at least one major hepatic vein. These major structures can be divided by hand suturing but in recent years the vast majority of liver surgeons have taken to using surgical staplers. These devices make the division of vascular pedicles quick and safe and although they are associated with increased cost they greatly reduce operating time.

The principle problem with operating on the liver is that it is a solid parenchymal organ. Its division is technically challenging and it is this fact that has made liver surgery the province of relatively small numbers of specialist surgeons. Numerous techniques are available to divide the parenchyma. Historically liver resections were performed by what was appropriately called a finger fracture technique, whereby the liver was literally crushed between fingers (Pamecha *et al*, 2009). This left some of the more robust vascular structures intact and these could be controlled and divided by other means. However this does not control small vessels or fragile hepatic veins from which most bleeding occurs. This brutal and imprecise technique was replaced and developed by a variety of crushing and clamping techniques using simple surgical instruments. These methods are still used although by observation most surgeons use one of the newer technologies. Indeed most surgeons would not contemplate operating on the liver now without technically advanced instrumentation. There are a large number of instruments (Pamecha *et al*, 2009), all of which have utility to some degree, which are marketed by various manufacturers. The issue that all of this instrumentation tries to deal with is the fact that the solid structure of the liver is permeated with blood vessels and bile ducts of varying size, which require to be closed to prevent bleeding or bile leakage. Personal observation suggests the most common of the technologies used by liver surgeons to be the ultrasound aspirator and argon diathermy. The ultrasound aspirator uses ultrasound to destroy the parenchymal component of the liver but with insufficient energy to completely destroy the fibrous vascular structure. With experience it is possible to isolate the smallest vessels within the liver substance and deal with these by other means. However, the instrument requires experience to use; although there is a degree of selection for blood vessels, the energy will indeed rupture these if used without care. The precision of dissection that this instrument allows is much greater than can be achieved by mechanical means alone. In addition when operating close to tumour the instrument itself produces a margin so that resection margins are often better than might appear on histological assessment (Konopke *et al*, 2008).

Argon diathermy is used to seal blood vessels. Argon is an inert gas and creating argon gas plasma enables high temperatures to be delivered to the tissues, sealing blood vessels but not causing charring as would be the case if air or another reactive gas were used (Primrose, 2002).

Although this combination of ultrasound aspirator and argon diathermy is perhaps the most commonly used combination, there is virtually an endless array of instruments now available, which some surgeons find satisfactory. The Hydrojet Cutter is a fine high-pressure jet of water, which essentially performs the same function as the ultrasound aspirator. Ultrasound shears can be used to divide the liver and seal the associated vessels. Diathermy/radiofrequency devices are also favoured by some surgeons. However as is usually the case with any form of surgery, the surgeon's skill and technique is more important than the instrumentation. This is borne out in a Systematic Review, which shows little difference between the various techniques save that the crush-clamp technique is most economical (Pamecha *et al*, 2009).

## Minimally invasive surgery

In the last few years there has been a great deal of interest in minimally invasive or laparoscopic liver surgery. The liver may not at first sight seem an ideal situation for laparoscopic surgery given that the resection specimen may be quite a significant size. There is however some features, which make laparoscopic liver surgery worth serious consideration. Most cases involve resection without reconstruction and this certainly is the case with the vast majority of resections for metastases. In laparoscopic surgery it is reconstructing bowel or blood vessels with a necessity of intra-corporeal suturing, which can make the procedure technically very challenging. Further, although the liver specimen can be large, it can be distorted relatively easily when placed within a suitable laparoscopic extraction bag. Even a right hepatectomy specimen can be removed through a relatively small low-abdominal incision.

To perform laparoscopic liver surgery a surgeon requires to be extremely experienced in managing the conventional open case in addition to having a high level of laparoscopic skill. Essentially the same technique that is used for open resection is adapted for the laparoscopic procedure and all of the technology used in open cases has been adapted for laparoscopic use. Control of vascular inflow to the liver is routine. The intra abdominal pressure associated with laparoscopy is certainly helpful in that it reduces bleeding from the hepatic veins. Unlike the normal open procedure the hepatic veins are divided last. Parenchymal transection is performed using the same instrumentation as for the open approach, although in our experience ultrasound shears are easier to use laparoscopically.

**Figure 2:**
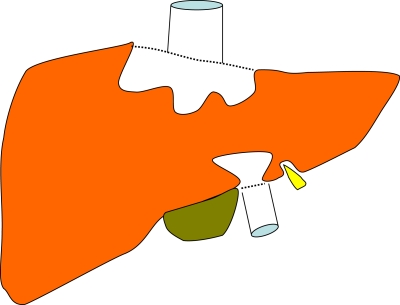
The areas of the liver most amenable to treatment by laparoscopic surgery. The grey area is accessible but those in white (mostly around the hepatic veins and the vena cava) much less so.

Laparoscopic liver resection is in general more technically demanding than the open approach. As such, complex resections around the centre of the liver are still best performed open as shown in Figure 2. The simplest areas to deal with laparoscopically are the most peripheral, segments 2 and 3, and 5 and 6 are ideal. In the HPB unit in Southampton, approximately 50% of liver resections are performed laparoscopically, with results that in the short term seem no different than open procedures in terms of resection margins and early tumour recurrence (Nguyen *et al*, 2009; Abu Hilal *et al*, 2010). Virtually all left sections (removal of segments 2 and 3) are performed laparoscopically (Abu Hilal and Pearce, 2008). These results are consistent with experience in other centres (Nguyen *et al*, 2009).

Laparoscopic liver surgery is unlikely to produce any significant benefit to patients in terms of cancer control. Indeed care must be exercised in adopting the technique as any reduction in cancer cure rate cannot be offset by the advantages. The main advantage for the patient is a much shorter hospital stay (Abu Hilal *et al*, 2010). With most surgery for metastatic disease the factor limiting patient's discharge is the large abdominal wound and consequent pain and immobility. Removing this factor in practice allows early discharge. If it can be shown that the oncological outcomes are the same (or better) then this is an advantage to the patient and also to the service.

Even when right hepatectomies are performed it is normally for lesions peripherally in the liver rather than centrally. This will undoubtedly change with more experience. Nonetheless, in the long term problems remain in dealing with the difficult situations, which involve intra-corporeal suturing. While this can clearly be accomplished, it is much more difficult than in the open case. In addition the angles may be unnatural for the operating surgeon by virtue of the port position, long straight instruments and lack of wrist movement at the tips. Robotic surgery may offer an advance here as the more natural hand movements can make some procedures possible that would be difficult laparoscopically. However, there is currently little published evidence to determine whether this will become a standard approach in minimally invasive liver resection.

## Techniques to increase the FLR

In recent years surgeons have examined ways of treating patients who would have an inadequate FLR after resection. It is known that if a liver resection leaves 25% or less of functioning liver the complication rate and the peri-operative mortality is high. In addition use of cytotoxic chemotherapy, which is known to cause significant liver injury, increases the complication rate after surgery (Nordlinger *et al*, 2008). It is now accepted that chemotherapy decreases the patient's capacity to withstand very large resections (Vauthey *et al*, 2006). As most patients now come to liver surgery having been treated with chemotherapy, this is clearly an important issue.

**Figure 3:**
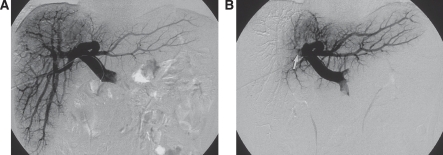
The technique of right portal vein embolisation. The catheter in introduced percutaneously into the main portal vein by the ipsilateral approach (**A**). The right portal vein is then ablated by embolising with a combination of tissue glue, microspheres and coils (**B**).

**Figure 4:**
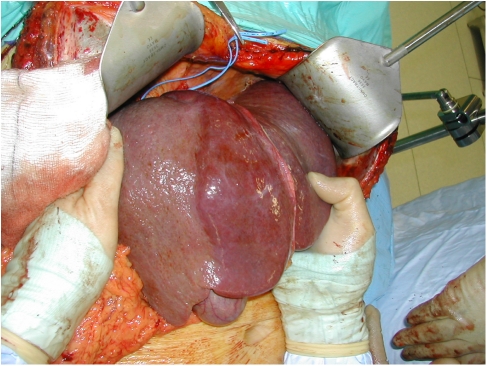
The liver at operation 6 weeks after portal vein embolisation. There is a clear line of demarcation in the line of the gall bladder and the inferior vena cava. The right liver is atrophic and shows signs of arterialisation.

There are two techniques increasingly used to treat such patients: first a staged resection and second portal vein embolisation (Figures 3 and 4). In the staged resection, one lobe of the liver is usually cleared of tumour or resected initially. Then after a period of recovery, usually around a month, the contralateral side is dealt with. In this time, regeneration and recovery of the initially treated lobe should occur. The other technique is portal vein embolisation. This was first described by Makuuchi (Makuuchi *et al*, 1990) in the context of hilar cholangiocarcinoma. It relies on the fact that if the portal vein to a lobe of liver is ablated, then there is atrophy of the corresponding liver and hypertrophy of the remainder. Essentially portal vein embolisation is used to cause atrophy of the right liver and hence cause hypertrophy in the left. If needed the branches to segment-4 of the liver can also be ablated, which leads to most hypertrophy occurring in segments 1, 2 and 3 (Hemming *et al*, 2003). This greatly increases the number of resections, which can be performed with safety. Surprisingly and unlike hepatic artery embolisation, portal vein embolisation produces virtually no symptoms in the patient. It therefore can be performed as a day case or with a very short stay. There is usually no significant disturbance to biochemically measured liver function and complications from the procedure appear to be very rare. Studies show, that in general terms the FLR volume can be increased by 30% or more (Hemming *et al*, 2003). Applying this technique can make the difference between an unsafe or impossible resection and one that can be accomplished with little morbidity.

In practice staged resection and portal vein embolisation are used together. A common scenario would be the clearance of metastases from the left liver followed by a right portal vein embolisation and then a second procedure to remove the right liver. Although the long-term utility of such procedures is not known, results in the medium term suggest that assuming clearance can be achieved they are no different from any other liver resection (Pamecha *et al*, 2008).

One area of innovation that has not become established is the *ante-situm* resection. In this procedure transplantation techniques are used to disconnect the liver from the vena cava. In addition the entire vascular pedicle to the liver can also be divided but in general terms this is inadvisable and unnecessary. The technique is used for disease around the hepatic veins. However the procedure is difficult, highly morbid and tumour outcomes are not particularly good. In fact it is likely that development of techniques to operate on the liver *in situ* that the *ante-situm* approach is actually seldom if ever required (Oldhafer *et al*, 2001).

## ‘Adjuvant’ surgery

The future of liver surgery for metastasis is likely to undergo a paradigm shift in the near future. At present the mantra for metastasis surgery is that unless a complete resection can be performed it should not be. It is now probably time to revaluate this approach. There are several reasons for this. First because of the advances in chemotherapy, colorectal cancer is beginning to resemble ovarian cancer where it is accepted that maximum surgical debulking has survival benefit (Tangjitgamol *et al*, 2009). In colorectal cancer it remains unproven whether maximum debulking surgery for metastatic disease is of benefit, but it seems likely. Oncologists have known for years that good response rates correlate with survival even when the best outcome is only a partial response (Giacchetti *et al*, 1999; Folprecht *et al*, 2005). Maximum surgical debulking can remove most of the disease and with a mortality that is no different from chemotherapy (Nordlinger *et al*, 2008). More recently the CLOCC trial, a randomised trial of ablation and chemotherapy *vs* chemotherapy alone, showed a survival benefit for ablation, even though the trial recruited extremely badly and had to be concluded as a randomised phase-II trial (Ruers *et al*, 2008). Thus the way forward seems to be in favour of a radical surgical approach in patients who respond to chemotherapy irrespective of whether the disease is completely removable. It is important, however, that this new concept is tested in a randomised trial as however safe the surgery it is likely that patients will spend some of their survival recovering from the operation. Such a trial is currently being developed within the UK NCRI.

## Concluding remarks

In conclusion, surgery for colorectal liver metastases is now well established. It has produced very significant benefits for patients, commonly now in combination with chemotherapy. Surgery itself may be nearing its technical limits but is likely to be applied to more patients due to success of other strategies, particularly newer chemotherapies and targeted therapies. What is most important for a patient is an expert multi-discipline approach to each individual problem.

## References

[bib1] Abu Hilal M, Pearce NW (2008) Laparoscopic left lateral liver sectionectomy: a safe, efficient, reproducible technique. Dig Surg 25: 305–308 18784413 10.1159/000155222

[bib2] Abu Hilal M, Underwood T, Zuccaro M, Primrose J, Pearce N (2010) Short- and medium-term results of totally laparoscopic resection for colorectal liver metastases. Br J Surg, doi:10.1002/bjs.7034 10.1002/bjs.7034 20474003

[bib3] Adam R, Wicherts DA, de Haas RJ, Ciacio O, Levi F, Paule B, Ducreux M, Azoulay D, Bismuth H, Castaing D (2009) Patients with initially unresectable colorectal liver metastases: is there a possibility of cure? J Clin Oncol 27: 1829–1835 19273699 10.1200/JCO.2008.19.9273

[bib4] Benoist S, Brouquet A, Penna C, Julie C, El Hajjam M, Chagnon S, Mitry E, Rougier P, Nordlinger B (2006) Complete response of colorectal liver metastases after chemotherapy: does it mean cure? J Clin Oncol 24: 3939–3945 16921046 10.1200/JCO.2006.05.8727

[bib5] Couinaud C, Delmas A, Patel J (1957) Le foie: Études anatomiques et chirurgicales. Masson: Paris

[bib6] Cresswell AB, Welsh FK, John TG, Rees M (2009) Evaluation of intrahepatic, extra-Glissonian stapling of the right porta hepatis *vs* classical extrahepatic dissection during right hepatectomy. HPB (Oxford) 11: 493–498 19816613 10.1111/j.1477-2574.2009.00083.x PMC2756636

[bib7] de Haas RJ, Wicherts DA, Flores E, Azoulay D, Castaing D, Adam R (2008) R1 resection by necessity for colorectal liver metastases: is it still a contraindication to surgery? Ann Surg 248: 626–637 18936576 10.1097/SLA.0b013e31818a07f1

[bib8] Finkelstein SE, Fernandez FG, Dehdashti F, Siegel BA, Hawkins WG, Linehan DC, Strasberg SM (2008) Unique site- and time-specific patterns of recurrence following resection of colorectal carcinoma hepatic metastases in patients staged by FDG-PET. J Hepatobiliary Pancreat Surg 15: 483–487 18836801 10.1007/s00534-007-1237-2

[bib9] Folprecht G, Grothey A, Alberts S, Raab HR, Kohne CH (2005) Neoadjuvant treatment of unresectable colorectal liver metastases: correlation between tumour response and resection rates. Ann Oncol 16: 1311–1319 15870084 10.1093/annonc/mdi246

[bib10] Fong Y, Bentrem DJ (2006) CASH (chemotherapy-associated steatohepatitis) costs. Ann Surg 243: 8–9 16371729 10.1097/01.sla.0000193599.57858.9b PMC1449964

[bib11] Giacchetti S, Itzhaki M, Gruia G, Adam R, Zidani R, Kunstlinger F, Brienza S, Alafaci E, Bertheault-Cvitkovic F, Jasmin C, Reynes M, Bismuth H, Misset JL, Levi F (1999) Long-term survival of patients with unresectable colorectal cancer liver metastases following infusional chemotherapy with 5-fluorouracil, leucovorin, oxaliplatin and surgery. Ann Oncol 10: 663–669 10442188 10.1023/a:1008347829017

[bib12] Goslin R, Steele Jr G, Zamcheck N, Mayer R, MacIntyre J (1982) Factors influencing survival in patients with hepatic metastases from adenocarcinoma of the colon or rectum. Dis Colon Rectum 25: 749–754 7172942 10.1007/BF02553304

[bib13] Guillou PJ, Quirke P, Thorpe H, Walker J, Jayne DG, Smith AM, Heath RM, Brown JM (2005) Short-term endpoints of conventional *vs* laparoscopic-assisted surgery in patients with colorectal cancer (MRC CLASICC trial): multicentre, randomised controlled trial. Lancet 365: 1718–1726 15894098 10.1016/S0140-6736(05)66545-2

[bib14] Hemming AW, Reed AI, Howard RJ, Fujita S, Hochwald SN, Caridi JG, Hawkins IF, Vauthey JN (2003) Preoperative portal vein embolization for extended hepatectomy. Ann Surg 237: 686–691; discussion 691–3 12724635 10.1097/01.SLA.0000065265.16728.C0 PMC1514515

[bib15] Konopke R, Kersting S, Makowiec F, Gassmann P, Kuhlisch E, Senninger N, Hopt U, Saeger HD (2008) Resection of colorectal liver metastases: is a resection margin of 3 mm enough?: a multicenter analysis of the GAST Study Group. World J Surg 32: 2047–2056 18521661 10.1007/s00268-008-9629-2

[bib16] Makuuchi M, Thai BL, Takayasu K, Takayama T, Kosuge T, Gunven P, Yamazaki S, Hasegawa H, Ozaki H (1990) Preoperative portal embolization to increase safety of major hepatectomy for hilar bile-duct carcinoma – a preliminary-report. Surgery 107: 521–527 2333592

[bib17] Morris E, Thomas J, Forman D, Quirke P, Cottier B, Poston GJ (2009) The need for a revised staging system of metastatic (M) colorectal cancer (CRC): evidence from a national perspective on survival following surgically treated (HPX) liver metastases [Abstract 4099]. J Clin Oncol ASCO Annu Meet Proc 27: 4099

[bib18] Mulier S, Ruers T, Jamart J, Michel L, Marchal G, Ni YC (2008) Radiofrequency ablation *vs* resection for resectable colorectal liver metastases: time for a randomized trial? Dig Surg 25: 445–460 19212117 10.1159/000184736

[bib19] Nguyen KT, Laurent A, Dagher I, Geller DA, Steel J, Thomas MT, Marvin M, Ravindra KV, Mejia A, Lainas P, Franco D, Cherqui D, Buell JF, Gamblin TC (2009) Minimally invasive liver resection for metastatic colorectal cancer: a multi-institutional, international report of safety, feasibility, and early outcomes. Ann Surg 250: 842–848 19806058 10.1097/SLA.0b013e3181bc789c

[bib20] Nordlinger B, Sorbye H, Glimelius B, Poston GJ, Schlag PM, Rougier P, Bechstein WO, Primrose JN, Walpole ET, Finch-Jones M, Jaeck D, Mirza D, Parks RW, Collette L, Praet M, Bethe U, Van Cutsem E, Scheithauer W, Gruenberger T (2008) Perioperative chemotherapy with FOLFOX4 and surgery *vs* surgery alone for resectable liver metastases from colorectal cancer (EORTC Intergroup trial 40983): a randomised controlled trial. Lancet 371: 1007–1016 18358928 10.1016/S0140-6736(08)60455-9 PMC2277487

[bib21] Oldhafer KJ, Lang H, Malago M, Testa G, Broelsch CE (2001) [*Ex situ* resection and resection of the *in situ* perfused liver: are there still indications?]. Chirurg 72: 131–137 11253671 10.1007/s001040051280

[bib22] Pamecha V, Gurusamy KS, Sharma D, Davidson BR (2009) Techniques for liver parenchymal transection: a meta-analysis of randomized controlled trials. HPB (Oxford) 11: 275–281 19718353 10.1111/j.1477-2574.2009.00057.x PMC2727079

[bib23] Pamecha V, Nedjat-Shokouhi B, Gurusamy K, Glantzounis GK, Sharma D, Davidson BR (2008) Prospective evaluation of two-stage hepatectomy combined with selective portal vein embolisation and systemic chemotherapy for patients with unresectable bilobar colorectal liver metastases. Dig Surg 25: 387–393 19033722 10.1159/000176063

[bib24] Pilkington SA, Rees M, Peppercorn D, John TG (2007) Laparoscopic staging in selected patients with colorectal liver metastases as a prelude to liver resection. HPB (Oxford) 9: 58–63 18333114 10.1080/13651820601150986 PMC2020775

[bib25] Primrose JN (2002) Treatment of colorectal metastases: surgery, cryotherapy, or radiofrequency ablation. Gut 50: 1–5 11772955 10.1136/gut.50.1.1 PMC1773068

[bib26] Rappeport ED, Loft A (2007) Liver metastases from colorectal cancer: imaging with superparamagnetic iron oxide (SPIO)-enhanced MR imaging, computed tomography and positron emission tomography. Abdom Imaging 32: 624–634 17710359 10.1007/s00261-007-9297-y

[bib27] Rees M, Tekkis PP, Welsh FK, O’Rourke T, John TG (2008) Evaluation of long-term survival after hepatic resection for metastatic colorectal cancer: a multifactorial model of 929 patients. Ann Surg 247: 125–135 18156932 10.1097/SLA.0b013e31815aa2c2

[bib28] Ruers T, van Coevorden F, Pierie J, Borel Rinkes I, Punt C, Ledermann J, Poston GJ, Bechstein W, Lentz M, Collette L, Nordlinger B (2008) Radiofrequency ablation (RFA) combined with chemotherapy for unresectable colorectal liver metastases (CRC LM): Interim results of a randomised phase II study of the EORTC-NCRI CCSG-ALM Intergroup 40004 (CLOCC) [Abstract 2012]. J Clin Oncol 26. ASCO Meeting Abstracts 20 May 2008: 401218711192

[bib29] Ruers TJ, Wiering B, van der Sijp JR, Roumen RM, de Jong KP, Comans EF, Pruim J, Dekker HM, Krabbe PF, Oyen WJ (2009) Improved selection of patients for hepatic surgery of colorectal liver metastases with (18)F-FDG PET: a randomized study. J Nucl Med 50: 1036–1041 19525451 10.2967/jnumed.109.063040

[bib30] Simmonds PC, Primrose JN, Colquitt JL, Garden OJ, Poston GJ, Rees M (2006) Surgical resection of hepatic metastases from colorectal cancer: a systematic review of published studies. Br J Cancer 94: 982–999 16538219 10.1038/sj.bjc.6603033 PMC2361241

[bib31] Tangjitgamol S, Manusirivithaya S, Laopaiboon M, Lumbiganon P (2009) Interval debulking surgery for advanced epithelial ovarian cancer: a Cochrane systematic review. Gynecol Oncol 112: 257–264 19017548 10.1016/j.ygyno.2008.09.041

[bib32] Tomlinson JS, Jarnagin WR, DeMatteo RP, Fong Y, Kornprat P, Gonen M, Kemeny N, Brennan MF, Blumgart LH, D’Angelica M (2007) Actual 10-year survival after resection of colorectal liver metastases defines cure. J Clin Oncol 25: 4575–4580 17925551 10.1200/JCO.2007.11.0833

[bib33] Vauthey JN, Pawlik TM, Ribero D, Wu TT, Zorzi D, Hoff PM, Xiong HQ, Eng C, Lauwers GY, Mino-Kenudson M, Risio M, Muratore A, Capussotti L, Curley SA, Abdalla EK (2006) Chemotherapy regimen predicts steatohepatitis and an increase in 90-day mortality after surgery for hepatic colorectal metastases. J Clin Oncol 24: 2065–2072 16648507 10.1200/JCO.2005.05.3074

[bib34] Wang WD, Liang LJ, Huang XQ, Yin XY (2006) Low central venous pressure reduces blood loss in hepatectomy. World J Gastroenterol 12: 935–939 16521223 10.3748/wjg.v12.i6.935 PMC4066160

[bib35] Wicherts DA, Miller R, de Haas RJ, Bitsakou G, Vibert E, Veilhan LA, Azoulay D, Bismuth H, Castaing D, Adam R (2008) Long-term results of two-stage hepatectomy for irresectable colorectal cancer liver metastases. Ann Surg 248: 994–1005 19092344 10.1097/SLA.0b013e3181907fd9

